# Evaluating the Potential Effect of Melatonin on the post-Cardiac Surgery Sleep Disorder

**Published:** 2015-07-03

**Authors:** Mehrnoush Dianatkhah, Padideh Ghaeli, Azita Hajhossein Talasaz, Abbasali Karimi, Abbas Salehiomran, Peyvand Bina, Arash Jalali, Saba Ghaffary, Nazila Shahmansouri, Shaghayegh Vejdani

**Affiliations:** 1 *Department of Pharmacotherapy, Faculty of Pharmacy, Tehran University of Medical Sciences, Tehran, Iran.*; 2 *Tehran Heart Centre, Tehran University of Medical Sciences, Tehran, Iran.*

**Keywords:** *Sleep disorders*, *Coronary artery bypass*, *Melatonin*

## Abstract

**Background**: Postoperative neurological injuries, including cognitive dysfunction, sleep disorder, delirium, and anxiety, are the important consequences of coronary artery bypass graft surgery (CABG). Evidence has shown that postoperative sleep disturbance is partly due to disturbed melatonin secretion in the perioperative period. The aim of this study was to evaluate the effect of melatonin on postoperative sleep disorder in patients undergoing CABG.

**Method: **One hundred forty-five elective CABG patients participated in a randomized double-blind study during the preoperative period. The patients were randomized to receive either 3 mg of melatonin or 10 mg of Oxazepam one hour before sleep time. Each group received the medication from 3 days before surgery until the time of discharge. Sleep quality was evaluated using the Groningen Sleep Quality Score (GSQS), and the incidence of delirium was evaluated by nursing records. Sleep quality and anxiety scores were compared before and after surgery through the Wilcoxon signed-rank test. The analysis of covariance (ANCOVA) and independent t-test were used to compare the sleep and anxiety scores between the groups. P values ≤ 0.05 were considered statistically significant**.**

**Results:** Totally, 137 patients at a mean age of 60 years completed the study (76% male). The analysis of the data showed that sleep was significantly disturbed after surgery in both groups. The patients in the Oxazepam group demonstrated significantly higher disturbance in their mean postoperative GSQS score than did their counterparts in the melatonin group (p value < 0.001). A smaller proportion of the participants experienced delirium in the melatonin group (0.06%) than in the Oxazepam group (0.12%); however, this difference was not statistically significant.

**Conclusion: **The result of the present study revealed that melatonin improved sleep in post-cardiac surgery patients more than what was observed with Oxazepam. Therefore, melatonin may be considered an effective alternative for Benzodiazepines in the management of postoperative sleep disorder.

## Introduction

Coronary artery bypass graft surgery (CABG) is one of the well-studied surgical modalities in the history of surgical procedures. This operation has been reported to be highly effective in the treatment of severe angina that is unresponsive to medical management or percutaneous coronary intervention.^        1 ^ There is a general agreement that CABG improves lifespan and prognosis in the postoperative years in patients with severe angina. Given the advances in cardiac surgery and low mortality rates, it is important to pay attention to the quality of life in postsurgical patients.^2^ In spite of improvements in surgical and anesthetic techniques, postoperative neuropsychiatric disturbances, including cognitive dysfunction, sleep disorder, delirium, and anxiety, are noted to be the important causes of postoperative morbidity and to be able to influence the outcome of treatment as well as long-term quality of life.^3^^, ^^4^ Sleep deprivations, including sleeplessness, low sleep efficiency, difficulty in falling asleep, and frequent nocturnal awakenings, have been reported by 39%–69% of patients who have undergone CABG during the first few postoperative weeks.^5^^, ^^6^ Evidence has shown that postoperative sleep disorder may be partly due to disturbed melatonin secretion in the perioperative period.^4^

Melatonin is a neurohormone originating from the amino acid, tryptophan, and is mainly secreted by the pineal gland into the blood stream and the cerebrospinal fluid. It possesses a circadian secretion pattern with a low blood concentration during the day and a high concentration at night.^7^ Most of the melatonin in blood is metabolized in the liver before being excreted into urine.^8^ Studies using radioactively-labeled melatonin have identified that melatonin exerts many of its physiological actions by interacting with two probable receptors: MT1 and MT2, which are located in the suprachiasmatic nucleus of the hypothalamus, pars tuberalis of the pituitary, hippocampus of the central nervous system (CNS), retina, cardiac blood vessel, and various peripheral organs.^7^^, ^^9^ The United States Food and Drug Administration has categorized melatonin as a dietary supplement for the treatment of insomnia.^10^ This amino acid affects sleep quality by reducing sleep latency and increasing total sleep duration without changing its architecture. The efficacy and safety of exogenous melatonin for the management of secondary sleep disorders has been confirmed in meta-analyses.^3^^, ^^11^ Based on the above-affirmed data, the present study sought to evaluate the effect of melatonin on postoperative sleep in patients undergoing CABG. 

## Methods

One hundred forty-five elective CABG patients participated in this randomized double-blind study during the preoperative period. All the patients signed informed consent forms. The study protocol was approved by the Ethics Committee for Human Research at Tehran University of Medical Sciences. Subjects taking psychiatric medications, CNS depressants, and hypnotic drugs or those with a history of suffering from any sleep disorder were excluded from the study. Patients were allocated to each group using the permuted-block randomization method(block stratified randomization software for Windows [version 6], Steven Piantadozi [2010]).The subjects received either 3 mg of melatonin (Melatonin, Nature Made, Canada, and USA) or 10 mg of Oxazepam (Sobhan, Tehran, Iran) one hour before assigned sleep time. Each group received the medication from 3 days before the operation until the time of discharge. All the patients in the study underwent their operation with the same technique (on-pump), performed by the same surgical team, and received the same cardiac medication regimen, comprising angiotensin-converting enzyme inhibitors, beta blockers, vasodilators, lipid-lowering agents, Aspirin, and Heparin, before and after the operation.

In this study, the sample size was estimated based on α of 0.05 and study power of 95%. Considering 30% loss of the cases and according to related studies with SD (1) = 3.55, SD (2) = 2.69, and d = 2.33, a sample size of 66 for each group was proposed.^12^

Sleep quality was evaluated using the Groningen Sleep Quality Score (GSQS). To our knowledge, the GSQS is the only questionnaire reported to evaluate the previous night's sleep quality. The GSQS consists of 15 questions about the previous night's sleep, answered with Yes or No.^13^^, ^^14^ The sum of this scale yields a generalized score of the previous night's sleep quality. A higher score in the GSQS means a more disturbed sleep during the previous night. Previous studies have shown the validity and reliability of the GSQS for sleep assessment.^15^ The subjects were asked to fill the 15-item Groningen List of Sleep Complaints (GLSC, Department of Biological Psychiatry, University Hospital of Groningen, the Netherlands; unpublished) in the morning early after waking up, four times during their hospitalization, and two times before and two times after surgery (days -6, -5, +3, and +5). The mean of the two scores before and after surgery was used for the comparison of sleep quality. Additionally, the patients were asked to describe the quality of their previous sleep and to verbalize those factors that may have resulted in their sleep disturbance such as pain and noise.

The Hamilton Anxiety Rating Scale (HAM-A) is a psychological questionnaire employed by clinicians to rate the severity of anxiety. This scale includes 14 items. Each consists of a number of symptoms, and the symptoms are graded on a scale of zero to four.^16^ All of these scores are utilized to calculate an overarching score that indicates the severity of a person’s anxiety.

The incidence of delirium was evaluated by using nursing records. The patients’ delirious state was assessed based on the clinical observations of trained nurses. 

The continuous variables are presented as mean with standard deviation (SD) and median with interquartile range (IQR) boundaries when the data were not normally distributed. The continuous variables were compared between the intervention groups using the student t or Mann-Whitney U test. The categorical variables are expressed through frequencies with percentages and were compared between the two intervention groups using the chi-square test. The sleep quality and anxiety scores were compared before and after surgery through the Wilcoxon signed-rank test. The analysis of covariance (ANCOVA) was used to compare the anxiety scores between the groups adjusting for their measures before surgery, and the independent t-test for sleep quality. P values ≤ 0.05 were considered statistically significant. IBM SPSS Statistics for Windows, version 20.0, (Armonk, NY: IBM Corp.) was applied to conduct the analyses.

## Results

In our study, 145 CABG patients were enrolled. Of this total, 8 (5.5%) patients were excluded from the trial due to postoperative complications. Therefore, the statistical analysis was performed on 137 patients: 66(48.2%) subjects in the melatonin group and 71 (51.8%) in the Oxazepam arm. There were no significant differences in the baseline characteristics of the included patients in terms of age, gender, and body mass index (BMI) (Table 1). The risk factors and perioperative parameters showed no significant differences between the groups except for dyslipidemia, pump time, cross-clamp time, total intensive care unit (ICU) hours, total ventilation time, and number of grafts (Table 1).All the patients had uncomplicated recovery from surgery. There was no significant difference (p value=0.436) between the melatonin and Oxazepam groups regarding the mean preoperative GSQS score (Table 2). The analyses revealed that sleep was significantly disturbed after surgery in both groups. The patients in the Oxazepam group demonstrated significantly higher disturbance in their mean postoperative GSQS score than did those in the melatonin group (p value < 0.001). A smaller proportion of the participants experienced delirium in the melatonin group (n = 4, 0.06%) than in the Oxazepam group (n = 9, 0.12%), but this difference was not statistically significant (p value = 0.187), (achieved power = 25.6%).

**Table 1:** Demographic, risk factors, and perioperative parameters

	Melatonin (n=66)	Oxazepam (n=71)	P Value
Demographic Variables			
Age (y)	60.03±10.21	61.70±9.86	0.331*
Male sex	53 (80.3)	52 (73.2)	0.329***
Body mass index (kg/m^2^)	27.09±4.55	28.04±3.60	0.175*
Risk Factors			
Family history	13 (21.0)	16 (25.4)	0.558***
Hyperlipidemia	43 (66.2)	32 (48.5)	0.041***
Hypertension	31 (47.7)	41 (62.1)	0.097***
Diabetes mellitus	21 (32.3)	22 (33.3)	0.901***
Smoking	13 (20.6)	10 (15.4)	0.439***
Baseline Lab Data			
Total cholesterol (mg/dl)	146.21±38.22	146.11±39.92	0.983*
High-density lipoprotein (mg/dl)	35.0 (29.0-41.0)	37.0 (30.0-45.0)	0.305**
Low-density lipoprotein (mg/dl)	79.0 (64.0-106.0)	87.0 (68.0-108.0)	0.620**
Triglyceride (mg/dl)	146.97±81.12	125.57±45.57	0.319**
Serum creatinine (mg/dl)	0.89 (0.77-1.01)	0.88 (0.76-0.98)	0.671**
Fasting blood sugar (mg/dl)	101.33±27.00	94.43±19.63	0.107*
Postoperative Parameters			
Sinus rhythm	62 (98.4)	65 (100.0)	0.115***
Atrial fibrillation	1 (1.6)	3 (4.6)	0.619***
Total ventilation Time (hr)	23.0 (18.8-42.2)	24.5 (22.1-46.3)	0.689**
ICU stay (hr)	23.0 (18.8-42.2)	24.5 (22.1-46.3)	0.024**
Intraoperative Parameters			
Intra-aortic balloon	1 (1.6)	1 (1.6)	0.999***
Blood transfusion	16 (25.4)	15 (23.1)	0.759***
Total grafts	4.0 (3.0-4.0)	3.0 (3.0-4.0)	0.041**
Pump time (min)	80.0 (65.0-94.5)	71.5 (50.0-90.0)	0.016**
Cross-clamp time (min)	47.5 (39.7-55.5)	42.5 (30.0-53.5)	0.016**
Inotrope	8 (12.7)	16 (24.6)	0.084***

Data are expressed as mean±SD and median (IQR boundaries) for the normally and non-normally distributed continuous variables; as numbers (percentages) for the categorical variables

* Independent samples t-test

** Mann-Whitney U test

*** Chi-square tes**t**

**Table 2:** Effect of melatonin on postoperative sleep, anxiety, and delirium

	Melatonin	Oxazeoam	P Value
Delirium			
After intervention	4 (6.1)	9 (12.7)	0.187*
Sleep			
Before intervention	2.0 (1.0-3.0)	2.0 (1.5-3.5)	0.436**
After intervention	2.5 (1.5-5.6)	8.0 (3.0-11.0)	< 0.001**
P Value	0.001***	< 0.001***	
Anxiety			
Before intervention	3.5 (2.0-6.2)	6.0 (2.0-10.0)	0.055**
After intervention	5.0 (3.7-8.2)	9.0 (5.0-13.0)	0.171****
P Value	0.013***	< 0.001***	

Variables are presented as n (%), mean with±SD, or median with interquartile range (IQR) boundaries

* Chi-square test

** Independent T-test

*** Wilcoxon signed-ranked test

**** Analysis of covariance

## Discussion

Sleep disturbance is common among patients undergoing CABG, especially during the first week of the postoperative period. Sleep disorders result in important impacts on morbidity, mortality, and quality of life.^5^ Sleep disturbances, including sleeplessness, low sleep efficiency, difficulty in falling asleep, frequent nocturnal awakenings with no identified stimuli, and early morning awakenings, have been frequently reported by post-CABG patients.^5^^, ^^17^ The absence of regular sleep cycles and rapid eye movement (REM) sleep during the early postoperative weeks has been noted in many studies. Sleep pattern disturbances have been reported to continue as late as 6 to 12 months after heart surgery.^17^^, ^^18^ The association between sleep deprivation and impaired human functioning was demonstrated in a meta-analysis of 19 studies.^19^ Self-reported sleep disturbance is also correlated with psychological and physical complaints, symptoms of cardiac illness, and number of disability days.^17^ Many factors are thought to be the cause of sleep disturbance in patients who have undergone CABG. These factors include environmental stimuli (e.g. noise and uncomfortable beds), individual characteristics (e.g. primary sleep disorder and comorbid health), nature of cardiac illnesses, and surgical complications (e.g. incisional pain, use of diuretics and resultant nocturia, dyspnea, and difficulty in finding the proper position to sleep).^5^^, ^^17^Although the above-mentioned factors have been suggested to disturb the patient’s sleep, evidence has shown that the frequency of self-reported sleep disturbance does not appear to change after these problems are resolved.^17^ It has been suggested that no statistically significant relationship exists between pain and sleep disturbance.^17^^, ^^18^ As was stated previously, many patients complain of frequent nocturnal awakenings without obvious external stimuli or sleeplessness without any identified reasons. Accordingly, there may be another underlying reason for postoperative sleep disturbances in post-CABG patients.^17^ There is a hypothesis stating that the disturbances in the concentration of melatonin may result in the sleep disturbances of post-CABG patients.^4^ Furthermore, decreased plasma melatonin concentrations have been documented during surgery and the postsurgical period in patients having undergone CABG.^4^^, ^^20^ Since clinical studies have shown the association between melatonin secretion and sleep deprivation, delirium, ICU psychosis, and other behavioral disorders, melatonin could be regarded as an important endogenous factor regulating the sleep pattern of post-CABG patients.^4^^, ^^21^ Many factors are likely to influence melatonin secretion in the perioperative period; these parameters may include factors associated with surgery and anesthesia (e.g. eyes closed during general anesthesia and the eye patch intensify the darkness) as well as medications. Benzodiazepines, which are frequently used as sedatives, can disturb the rhythm of melatonin secretion by acting on the complex of the gamma-amino butyric acid receptor. Meanwhile, investigations have shown that narcotics may suppress the nocturnal secretion of melatonin. Beta blockers are another class of medications that may inhibit melatonin secretion by acting on adrenergic receptors located in the pineal gland. These medications are commonly used in the perioperative time and can be considered the main factors that can influence disturbed melatonin secretion.^4^^, ^^20^^,^^21^ Moreover, it seems that reduced plasma melatonin concentrations reflect the consumption of melatonin as an antioxidant to neutralize the free radicals induced by the surgical procedure.^22^

The present study aimed to investigate the effect of melatonin on sleep quality, anxiety, and incidence of delirium in patients undergoing CABG. To the best of our knowledge, this is the first study of its kind to evaluate the effect of melatonin on the prevention of postoperative sleep disorder. The result of this double-blind randomized clinical trial revealed that 3 mg of melatonin taken one hour prior to bedtime could improve sleep in post-cardiac surgery patients in comparison with Oxazepam. As is shown in Table 2, while sleep was significantly disturbed after surgery in both groups, the patients in the melatonin group demonstrated significantly milder disturbance in their mean postoperative GSQS score when compared with those in the Oxazepam group; this difference constituted statistical significance (p value < 0.001). This finding can be interpreted as a partial compensation for postoperative sleep disturbance conferred by the administration of melatonin. The underlying mechanism of sleep disturbance after CABG is somewhat due to a decrease in melatonin secretion during the early postoperative period. Therefore, it seems that modifying the melatonin level by adding exogenous melatonin to the natural circadian hormone can result in the amelioration of sleep disturbance. Postoperative complications, including nocturia due to diuretic use, incisional pain, and discomfort due to sleep positioning, have been frequently reported as the main causes of sleep disturbances in the early postoperative period. Some other patients in our study suffered sleep problems without any known cause. Although the incidence of delirium with melatonin was reported to be half of that with Oxazepam (6% vs. 12%), the difference was not statistically significant, which can be the result of the inclusion of a small sample. In general, the neuropathophysiology of delirium is poorly understood. There is some evidence in the literature that supports the use of melatoninin the treatment of postoperative delirium. Two mechanisms have been reported to justify the role of melatonin in decreasing the incidence of delirium. Since sleep disturbance is one of the key elements in inducing delirium,^23^^, ^^24^ the first mechanism considers the administration of exogenous melatonin in order to prevent delirium through improving postoperative sleep deprivations.^25^ The second mechanism is explained under the “tryptophan theory”. Tryptophan is converted to serotonin and then to the hormone, melatonin. All the three neurotransmitters are involved in the development of delirium.^4^^, ^^26^ Lewis and Barnett^26^ hypothesized that the administration of exogenous melatonin might prevent delirium by inhibiting the breakthrough of both serotonin and tryptophan by the feedback mechanism. Al-Aamaet al.^27^ demonstrated that a low dose of exogenous melatonin, given nightly to elderly patients in acute care settings, might be a potential protective agent against delirium. Hanania and Kitain^28^ reported the successful use of melatonin in the treatment of severe postoperative delirium unresponsive to Benzodiazepines or antipsychotics. Clinical studies have indicated that the anxiety level is usually increased just before the surgical procedure but drops rapidly afterward. However, there are reports that some patients would suffer high anxiety levels for a long time after surgery.^2^^, ^^29^ Melatonin has been shown to exert some anxiolytic properties, rendering it an alternative substitute for Oxazepam in practice.^29^^-^^31^ In contrast to other common hypnotics, melatonin does not seem to adversely affect the cognition or respiratory system of the patients receiving this medication.^12^^, ^^27^ In addition to its psychological benefits, melatonin exerts many cardiovascular and protective effects in patients undergoing CABG. Thanks to its antioxidant activity, melatonin can also be protective against reperfusion injury during surgery.^32^^, ^^33^ With regard to safety issues, melatonin does not attenuate dynamic cardiovascular and cerebrovascular responses to acute hypotension.^34^ Additionally, in contrast to conventional hypnotics, this drug does not worsen nocturnal hypoxemia or ventilator responses in patients with underlying respiratory problems.^12^^, ^^35^ Therefore, patients undergoing CABG may benefit more from melatonin than from Oxazepam as a sedative.

Our study encountered some limitations. Although subjective criteria, also used in our study, seem to be preferred over polysomnography when differentiating patients with insomnia (in accomplishing trials), actinography and polysomnography are considered to be very useful in evaluating sleep quality in detail. Therefore, information such as sleep latency, total sleep time, nap time (napping episode), nocturnal frequency of awaking, duration of awaking, and different sleep phases, which can be achieved more accurately by utilizing actigraphy and polysomnography, was not documented in our study.^5^ There was no placebo group in this study, which may adversely affect the results, and can be mentioned as a limitation of the study.

## Conclusion

In conclusion, it can be hypothesized that melatonin may be a safe and effective alternative in the management of postoperative sleep disorder. Neuropsychological injuries, including cognitive dysfunction, sleep disorder, delirium, and anxiety, are the important causes of postoperative morbidity and can influence the treatment outcome and long-term quality of life. Further controlled trials with larger numbers of patients are recommended to confirm the results of this study.

## Notes:

***This paper should be cited as:*** Dianatkhah M, Ghaeli P, Talasaz AH, Karimi AA, Salehiomran A, Bina P, Jalali A, Ghaffary S, Shahmansouri N, Vejdani S. Evaluating the Potential Effect of Melatonin on the post-Cardiac Surgery Sleep Disorder. J TehUniv Heart Ctr 2015;10(3):122-128.
